# Draft Genome Sequence of the Ascomycete *Phaeoacremonium aleophilum* Strain UCR-PA7, a Causal Agent of the Esca Disease Complex in Grapevines

**DOI:** 10.1128/genomeA.00390-13

**Published:** 2013-06-27

**Authors:** Barbara Blanco-Ulate, Philippe Rolshausen, Dario Cantu

**Affiliations:** Department of Viticulture and Enology, University of California, Davis, Davis, California, USAa; Department of Plant Sciences, University of California, Davis, Davis, California, USAb; Department of Botany & Plant Sciences, University of California, Riverside, Riverside, California, USAc

## Abstract

Grapevine infections by *Phaeoacremonium aleophilum* in association with other pathogenic fungi cause complex and economically important vascular diseases. Here we present the first draft of the *P. aleophilum* genome sequence, which comprises 624 scaffolds with a total length of 47.5 Mb (L_50_, 45; N_50_, 336 kb) and 8,926 predicted protein-coding genes.

## GENOME ANNOUNCEMENT

The esca disease complex of grapevines refers to five syndromes: brown wood-streaking, petri disease, young esca, esca, and esca proper (1), which are caused by the ascomycetes *Phaeoacremonium aleophilum* W. Gams, P. W. Crous, M. J. Wingfield, and L. Mugnai (teleomorph, *Togninia minima*), *Phaeomoniella chlamydospora*, and other fungal species (1–3). Disease symptoms include, internally, wood discoloration, streaking, and vascular necrosis (3) and, externally, leaf chlorosis and necrotic stripes, berry black spots, decline in vigor and yield, and in severe cases plant death (3, 4).

The effective colonization of the host tissues appears to depend on *P. aleophilum* competence to produce phytotoxic metabolites (5, 6), overcome host preformed and inducible barriers (7, 8), degrade the plant cell wall (CW) (8, 9), and cooperate with other pathogens during infection (1, 3).

*P. aleophilum* strain UCR-PA7 was collected from the margin of a grapevine (*Vitis vinifera* cv. “Thomson”) wood canker in a commercial vineyard (Fresno County, CA) in October 2011. UCR-PA7 was hyphal-tip purified and taxonomically characterized using DNA markers (10). DNA was extracted using a modified cetyltrimethylammonium bromide (CTAB) protocol (11) and sequenced to an estimated median coverage of 103× using the Illumina HiSeq2000 platform. Using CLC Genomics Workbench-v6.0, we assembled 99% of the 4.7 × 10^7^ trimmed (*Q* ≥30) and contaminant-filtered reads into 624 scaffolds (N_50_, 336 kb; L_50_, 45; G+C content, 49.66%; gaps, 83 kb) with a total length of 47.5 Mb. Assembly parameters were optimized based on the degree of completeness of the gene space estimated with CEGMA (12). The UCR-PA7 genome was estimated to be >97% complete based on the mapping of 248 low-copy and conserved core eukaryotic genes (CEGs) (12).

The gene finder Augustus (13) was trained using the CEG structures determined by CEGMA and identified *ab initio* 8,926 complete protein-coding genes on repeat-masked scaffolds (RepeatMasker [14]). A total of 97% of these genes have homologs in other ascomycetes (BLASTp, E value ≤10^–3^). We identified 658 potentially secreted proteins (SignalP-v4.0 [15]), of which at least 23% consist of putative plant CW-degrading enzymes based on homology to proteins in the CAZy database (16). Among these, 17 cellulases (GH3s, GH5s, GH6s, GH7s, and GH45s), 12 hemicellulases (GH10s, GH11s, GH31s, GH29s, GH67s, and GH115s), 21 pectin-degrading enzymes (GH28s, GH78s, PL1s, PL3s, PL4s, PL9s, CE8s, and CE12s), 12 callose-degrading enzymes (GH55s), and 1 cutinase (CE5) might play important roles during tissue colonization and systemic infection.

We also detected 79 cytochrome P450 monooxygenases, 2 laccases, and 2 lignin peroxidases, supporting the ability of *P. aleophilum* to degrade lignocellulose (2, 3, 8). However, the number of putative lignin-degrading proteins in the *P. aleophilum* genome is smaller than that in other wood-decay fungi previously described (e.g., *Neofusicoccum parvum*, 212 P450s [17]; *Eutypa lata*, 205 P450s [18]; and *Phanerochaete carnosa*, 266 P450s [19]), which suggests that synergism with other vascular pathogens during plant infection may favor the effective breakdown of lignified tissues (4, 8).

### Nucleotide sequence accession numbers.

This Whole-Genome Shotgun project has been deposited at DDBJ/EMBL/GenBank under the accession no. AORD00000000. The version described in this paper is the first version, AORD01000000.
